# Influencing mechanism of coal miners’ safety compliance: A chain mediating model

**DOI:** 10.3389/fpsyg.2022.942038

**Published:** 2022-12-08

**Authors:** Yongzhan Li

**Affiliations:** Education College, Xuchang University, Xuchang, China

**Keywords:** job insecurity, safety compliance, emotional exhaustion, mind wandering, coal miners

## Abstract

The influence that job insecurity has on employees’ safety psychology and behavior has been identified in many empirical studies, but few of these examine the influencing mechanism of job insecurity on coal miners’ safety behaviors. In the de-overcapacity circumstances of coal production in China, using the strength model of self-control and conservation of resources theories, a chain mediating model was constructed to determine the relationships between job insecurity, emotional exhaustion, mind wandering, and safety compliance among coal miners. Data were collected from 447 coal miners working in three coal mines of Henan Pingdingshan Coal Industry Group. It was found that job insecurity negatively affected safety compliance, and emotional exhaustion and mind wandering played a chain mediating effect in the relationship between job insecurity and miners’ safety compliance, along three specific paths. This study helps advance the understanding of the internal mechanisms of coal miners’ job insecurity and how this affects individual safety performance. It also provides empirical evidence that managers can use effectively intervene in coal miners’ safety performance.

## Introduction

As China’s economic development enters its new normal, ecological and environmental constraints are increasing, growth in energy demand is slowing, and coal overcapacity is becoming increasingly common (China National Energy Administration, 2016). To build an intensive, safe, efficient, and greener modern coal industry, China has been accelerating reduction of backward coal production capacity (i.e., implementing de-overcapacity) in recent years. The number of operating coal mines in China fell from about 10,800 in 2015 to about 5,300 in 2020, or a closure of more than half (Huajing Industrial Research Institute, 2022). Accordingly, the number of coal miners decreased by more than 30%. Meanwhile, progress has been made in the restructuring of the coal industry and in the management of safe production. In 2020, China’s mortality rate of per 1 million tons of coal production dropped to 0.054, approaching the level of those high-income countries (Yu and Li, 2020). However, as the largest coal producer in the world, China’s total number of coal mine accidents is still high, and mine safety remains a concern. Many researchers believe that human failings or errors cause most mining accidents (Lawrence, 1974; Raath, 1993; Hofmann et al., 1995). It has been also documented that unsafe behavior is responsible for 80 to 95% of workplace accidents, with human error often to blame (Paul and Maiti, 2005; Masia and Pienaar, 2010). Human error can usually be traced to employees who do not strictly comply with safety regulations to perform production activities: accordingly, many accidents can be avoided if the safety regulations are strictly followed (Hofmann et al., 1995). Therefore, compliance with safety regulations is key to avoiding coal mine accidents and improving safety performance. Of course, in addition to the negative impact of the national policy of de-overcapacity on coal miners’ psychology, the structural, i.e., organizational and industrial, causes of human error at the individual level should not be ignored. Previous studies have found that a range of organizational factors can affect the work of coal miners and lead to accidents. These include excessive workloads that do not allow adequate time for sleep, incomplete systems of quality inspection and acceptance, inappropriate technical solutions, unclear divisions of responsibility and weak accountability systems (Zhao, 2007; Yu and Li, 2020).

In China, most coal miners come from poor rural areas, where mining is the main source of income for their families. At present, the world economy is at risk of recession due to the combined impact of the coronavirus disease 2019 (COVID-19) pandemic and the ongoing trade war. In addition, in recent years, the large-scale wave of coal mine closures directly caused by the de-overcapacity initiative has increased concerns among China’s coal miners in relation to the continuity and stability of their jobs (Yi and Li, 2021). In view of this, the job insecurity of coal miners in China and its influence deserve serious attention and research.

Job insecurity can have a range of impacts on employees’ psychology and behavior. Research shows that job insecurity has a significant predictive effect on employees’ safety motivation, safety performance, industrial accidents, organizational commitment, turnover intention, emotional exhaustion, and work engagement (Probst and Brubaker, 2001; Sender et al., 2017). Safety performance is an independent field within work performance, including two components: safety compliance and safety participation (Griffin and Neal, 2000). Safety compliance is generally considered a core safety activity for keeping the workplace safe; it refers to employees’ compliance with safety standards, procedures, legal obligations, and requirements (e.g., wearing protective equipment; Griffin and Neal, 2000). Employees who do not follow specific safety rules or procedures are considered to have no safety compliance or to violate safety rules (Jiang et al., 2010). Violations can lead to workplace accidents, physical injuries, and socio-economic costs (Neal and Griffin, 2006; Jiang et al., 2010). Safety participation includes voluntary safety-related activities such as helping colleagues, eliminating hidden dangers, and voluntarily participating in safety awareness dissemination, in a similar way to organizational citizenship behavior (Griffin and Neal, 2000). Some research teams internationally have focused on the impact of job insecurity on safety performance for more than 20 years and have identified some possible mechanisms (Probst and Brubaker, 2001; Jiang and Probst, 2016). However, research in China has only recently begun to tackle this theme, and the amount of work that has been conducted remains very limited (Yu and Li, 2020). In view of the core role that safety compliance plays in safety performance, based on a literature review and from the perspective of psychological resources, this study introduced two variables, namely, emotional exhaustion and mind wandering, to explore the impact of job insecurity on safety compliance among China’s coal miners and its mechanism.

### Job insecurity and safety compliance

Perceived job insecurity has increasingly drawn the attention of researchers (Wilczyńska et al., 2020). Job insecurity refers to “a perceived threat to the continuity and stability of employment as it is currently experienced” (Shoss, 2017, P.1941). Although job insecurity is not the same as actual job loss, concerns regarding job continuity and stability can cause persistent stress experiences for employees and seriously affect their physical and mental health (Cheng and Chan, 2008). As a continuous stressor, job insecurity has been negatively linked to employees’ work engagement, well-being, and mental health (Ashford et al., 1989; Dekker and Shaufeli, 1995; Probst and Brubaker, 2001; Wilczyńska et al., 2020). Long-term job insecurity can lead to a deterioration in employees’ attitudes toward their organization and work, such as strong turnover intentions and poor organizational commitment (Hellgren et al., 1999; Probst and Brubaker, 2001). In addition, job insecurity is closely related to job dissatisfaction and unsafe behavior (Probst and Brubaker, 2001; Rundmo and Iversen, 2007). Therefore, in the mining industry, job insecurity cannot be ignored. Research has shown that when miners perceive a threat to their job continuity and stability, they will experience negative emotions, worsened work engagement (Wang et al., 2015), and poor job performance (Hsieh and Huang, 2017). In addition, miners’ perceived job insecurity can predict their absenteeism, turnover, and safety violations (Kinnunen et al., 2014; Lee and Jeong, 2017). A survey of 500 coal miners in China showed that job insecurity not only led to miners’ emotional exhaustion, but also reduced their attention to safety and increased the risk of accidents in downhole operations (Li et al., 2019).

In the job security and safety model developed by Neal et al. (2000), safety compliance describes the extent to which employees comply with safety procedures, safety standards, and legal obligations and requirements to carry out their work in a safe manner. Campbell (1992) noted that any activity incorporates a common reflection of individual skills, knowledge, and motivation. Therefore, employees’ motivation should be closely related to their safety behavior. Among the theories of motivation, Maslow’s theory of the hierarchy of needs is very well known (Maslow, 1943). According to this, physiological and safety needs are basic needs and cannot be ignored. If these needs are not guaranteed, survival, health, and the pursuit of other advanced needs can be seriously affected. Therefore, job insecurity can have a serious impact on employees’ achievement motivation and behavior. So far as safety behavior is concerned, empirical studies have shown that job insecurity and stress negatively affect safety compliance (Ashford et al., 1989; Probst and Brubaker, 2001). For example, in two cross-sectional studies and one longitudinal study, Probst and Brubaker (2001) explored the relationship between job insecurity and safety outcomes. Their results indicated that employees with higher job insecurity reported lower safety motivation and compliance, which in turn were associated with higher levels of workplace accidents. More recently, in South Africa, Masia and Pienaar (2010) investigated a convenience sample of 158 gold miners and found that both work stress and job insecurity were negatively correlated with safety compliance. Based on this literature, we propose the following hypothesis:

*H*1: Job insecurity negatively predicts safety compliance among coal miners.

### Mediating role of emotional exhaustion

To explore the influencing mechanism of employees’ job insecurity on safety behaviors, this study focused on the important psychological phenomenon of emotional exhaustion. This is a state in which one feels depleted of emotional resources at work. It is the most prominent aspect of job burnout and reflects a state of tension at work (Maslach et al., 2001). Some studies have found that job insecurity positively predicts emotional exhaustion in the workplace (Piccoli and Witte, 2015; Öztürk et al., 2017).

Recent studies have found that emotional exhaustion plays a mediating role in the relationship between job insecurity and certain negative outcomes. For example, Lawrence and Kacmar (2017) found that that emotional exhaustion partially mediates the impact of job insecurity on unethical employee behavior. Li et al. (2019) showed that coal miners’ emotional exhaustion partially mediated the relationship between job insecurity and attention to safety. Regarding the relationship between emotional exhaustion and safety behaviors, Yuan et al. (2020) investigated a sample of 159 workers at a chemical product manufacturing company. The results showed that emotional exhaustion was a mediator in the link between abusive supervision and safety behaviors. In addition, more recently, Balducci et al. (2021) found that emotional exhaustion negatively predicts employee work performance. Given safety performance (behaviors) as an extension of work performance, the findings of Balducci et al. (2021) undoubtedly demonstrate something important about the relationship between emotional exhaustion and miners’ safety behaviors. Few empirical studies have directly explored emotional exhaustion as a specific mechanism in the relationship between miners’ job insecurity and their safety behaviors. However, certain theoretical perspectives and research findings have provided reasons to explore the specific mechanisms in detail. Following the strength model of self-control (Baumeister et al., 1998), successful self-control is considered to depend on adequate resources for self-control. However, such resources are limited, and any self-control behavior consumes them, leading to self-depletion. It is important to note that persistent self-depletion can lead to insufficient levels self-control resources, which in turn negatively affects subsequent self-control actions. In the workplace, failures of self-control are often accompanied by an increase in negative work behaviors, such as deviant or unethical behaviors (Lanaj et al., 2014; Welsh and Ordóñez, 2014). According to the strength model of self-control, exercising self-control in response to stress drains psychological resources. Previous studies have suggested that emotional exhaustion is an important indicator of resource depletion in self-control (Wheeler et al., 2013; Whitman et al., 2014). Employees who have insufficient self-control resources due to having to cope with stress can experience emotional exhaustion, which is detrimental to behavioral and emotional self-control (Schmeichel and Baumeister, 2004), and can easily lead to harmful or unethical workplace behaviors (Whitman et al., 2014). Some researchers have found that the execution of safety behaviors depends on sufficient self-control resources, so the strength model of self-control can be used to partly explain the occurrence of unsafe behaviors and occupational injuries (Dai et al., 2015; Kao et al., 2016). Dai et al. (2015) suggested that constant work stress depletes employees’ self-control resources, thus reducing their level of safety compliance. For coal miners, their safety compliance is reduced when their self-control resources are continuously drained in coping with the stress arising from job insecurity, causing them to fail to effectively overcome unsafe behavioral impulses. Accordingly, we hypothesize:

*H*2: Emotional exhaustion mediates the relationship between job insecurity and safety compliance among coal miners.

### Mediating role of mind wandering

Executive functioning is necessary for consistent, efficient, and productive performance, and lapses of attention are detrimental across performance-based domains (Feldman et al., 2016). One phenomenon of attention lapses is mind wandering, which refers to an attentional shift away from a primary task or ongoing event in the external environment toward internally generated task-unrelated thoughts or feelings (Smallwood and Schooler, 2015). Mind wandering has been studied in a variety of contexts, including in standard laboratory tasks, reading, and during routine, everyday activities (Smallwood and Schooler, 2015). Mind wandering occurs when an individual’s attention is diverted from the current task and situation to internal thoughts and feelings unrelated to the current task (Dane, 2018). It is essentially daydreaming, a manifestation of attentional decay (Kinnunen et al., 2014). During the period of mind wandering, an individual’s attention shifts from external perceptual inputs to internal processing. This can easily result in information coding errors (Smallwood et al., 2003), which in turn affect the individual’s perception of the outside world (Schooler et al., 2011), and leads to further declines in external tasks performance (Christoff et al., 2009). Studies have shown that when people perform a task that requires concentration, mind wandering is often associated with negative outcomes, such as learning difficulties, accidents, injuries, and workplace dysfunction (Mooneyham and Schooler, 2013).

Individuals who suffer from severe mind wandering can easily be distracted by information outside of the task that they are performing. This is closely related to lapses in attention to safety. Among the influencing factors of mind wandering, emotional exhaustion has been of concern to many researchers. From the perspective of an individual’s mental state, Mcvay and Kane (2009) emphasized that emotional exhaustion could increase the incidence of mind wandering. The more severe the emotional exhaustion is, the less attention one has at one’s disposal. A survey of miners in China showed that emotional exhaustion negatively predicted miners’ attention to safety (Niu et al., 2016). For coal miners, the threat of job insecurity can consume their mental resources, make them physically and mentally tired, and make it difficult for them to focus on their current task, thus leading to mind wandering. This is not conducive to making correct judgments and decisions in relation to a target task and can lower the level of operation execution (Barron et al., 2011), thus further negatively affecting safety compliance (Li and Li, 2021). Given these findings, we put forth the following hypotheses:

*H*3: Emotional exhaustion positively predicts mind wandering among coal miners.

*H*4: Mind wandering mediates the relationship between job insecurity and safety compliance among coal miners.

**Figure 1:**
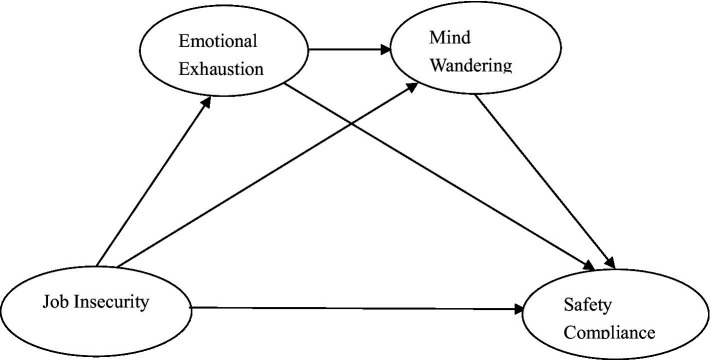
Research model.

Correspondingly, a chain mediation model based on these hypotheses is shown in Figure 1.

The literature review indicates that, although some studies have explored the impact of job insecurity on safety compliance among miners, the internal mechanism of the relationship between the two variables still needs close study. Few studies have investigated emotional exhaustion and mind wandering as mediating variables between job insecurity and safety compliance among miners. To address this research gap, drawing on the relevant literature, this study integrated emotional exhaustion, mind wandering, job insecurity, and safety compliance into a chain mediation model, obtained data through a questionnaire survey of Chinese coal miners, and tested the degree of fit between the chain mediation model and the data. We expect that the results of this study will deepen our understanding of the internal mechanism of job insecurity affecting safety compliance, and to provide empirical reference for the implementation of coal safety accident intervention and promotion of safety production.

## Materials and methods

### Sample and procedures

The data in this study were drawn a survey of miners conducted in the Henan Pingdingshan Coal Industry Group’s No. 1, No. 2, and No. 3 mines (each with a designed production capacity of over 4 million t/a). Data-collection occurred in May 2021. Ethics approval was obtained from Xuchang University Ethics Committee. In total, 500 questionnaires were distributed, with the assistance of the mining group’s human resources management department. A total of 459 miners completed the survey. Participants usually completed the surveys during their shift break from work, and the other participants completed the surveys while on leave. All procedures were conducted in accordance with the ethical standards of the 1964 Declaration of Helsinki. All participants were informed of the purposes and procedures of the study and provided written informed consent to participate. After removing questionnaires with significant missing data or an obvious answering tendency (for example, the respondent selected option “A” for more than five consecutive questions), our final sample size was 447 miners, and the effective return rate was 89.4%. The respondents aged between 23 and 57 years (32.42 ± 3.25 years), with work experience ranging from 1 to 31 years (9.35 ± 2.46 years). The other information of the sample is shown in Table 1.

**Table 1:** Descriptive statistics and correlation analysis of main variables (*N* = 447).

	*M*	SD	1	2	3	4
Job Insecurity	3.52	0.89	1			
Emotional Exhaustion	2.85	1.72	0.45^***^	1		
Mind Wandering	2.52	0.34	0.36^***^	0.43^***^	1	
Safety Compliance	2.33	0.45	−0.47^***^	−0.56^***^	−0.50^***^	1

^***^p < 0.001.

### Measures

We assessed job insecurity using a seven-item job insecurity scale developed by Hellgren et al. (1999). Each item was rated on a 5-point Likert scale ranging from 1 (strongly disagree) to 5 (strongly agree). A sample item is “I feel uneasy about losing my job in the near future.” This scale is widely used in the Chinese context and has good reliability and validity (Liu et al., 2021). In this study, the Cronbach’s α coefficient of the scale was 0.81.

Emotional exhaustion was measured with five items adopted from the Job Burnout Scale developed by Maslach et al. (1996). Participants evaluated their work status over the previous month using a 5-point Likert scale (ranging from 1 “never” to 5 “every day”). A sample item is “I feel burned out from my work.” In this study, the Cronbach’s α coefficient of the scale was 0.84.

Mind wandering was evaluated using the three-item Mind Wandering Frequency Questionnaire (Wang and Song, 2011). A sample item is “I find it hard to keep myself focused.” Participants were asked to rate items on a 5-point Likert scale (i.e., never, rarely, sometimes, often, and always). In the current study, the Cronbach’s α coefficient of the scale was 0.86.

The three-item Safety Compliance (SC) subscale of the Safety Behavior scale developed by Neal and Griffin (2006) was adopted to evaluate miners’ safety compliance. A 5-point Likert scale was used (ranging from 1 “strongly disagree” to 5 “strongly agree”). A sample item is “I use all the necessary safety equipment to do my job.” In this study, the Cronbach’s α coefficient of the scale was 0.79.

To be clear, total scores for all scales were computed by averaging scores.

### Data processing and analysis

SPSS 21.0 and AMOS 21.0 were used for data processing and analysis. Statistical methods included descriptive statistics, correlation analysis, exploratory factor analysis, and structural equation model analysis. Structural equation model is a statistical method to analyze the relationship between variables based on the covariance matrix of variables, and it is an important tool for multivariate data analysis. Structural equation model is a perfect combination of traditional path analysis and factor analysis. Traditional statistical methods cannot deal with latent variables effectively, while structural equation model can deal with latent variables and their indicators simultaneously. Different from traditional regression analysis, structural equation analysis can deal with multiple dependent variables at the same time, and can compare and evaluate different theoretical models. Structural equation model can also analyze complex mediation models, conduct statistical analysis on multiple competing models, and then decide which model is the most desirable according to how well each model fits the sample data.

### Control and test of common method bias

The data in this study were all collected through participants’ self-reports, which may lead to serious common method bias (Podsakoff et al., 2003). To reduce this risk, we took certain measures in the investigation, such as anonymizing the participants and providing reverse scoring for some items. To test the level of common method bias, Harman’s single factor test was used. The results showed that there were nine factors with eigenvalues greater than 1, explaining a total of 62.6% variance. The variance explained by the first factor was just 18.64%, far less than the threshold of 40% (Podsakoff et al., 2003), indicating that the common method bias of this study was within an acceptable range.

## Results

### Descriptive statistics and correlation analyses

The descriptive statistics and results of the correlation analyses of the main variables in this study are shown in Table 1. It was found that job insecurity was negatively correlated with safety compliance (*r* = −0.47), and positively correlated with emotional exhaustion (*r* = 0.45) and mind wandering (*r* = 0.36). Emotional exhaustion was positively correlated with mind wandering (*r* = 0.43). Both emotional exhaustion and mind wandering were negatively correlated with safety compliance (*r* = −0.56 and *r* = −0.50, respectively).

### Hypothesis test

**Figure 2:**
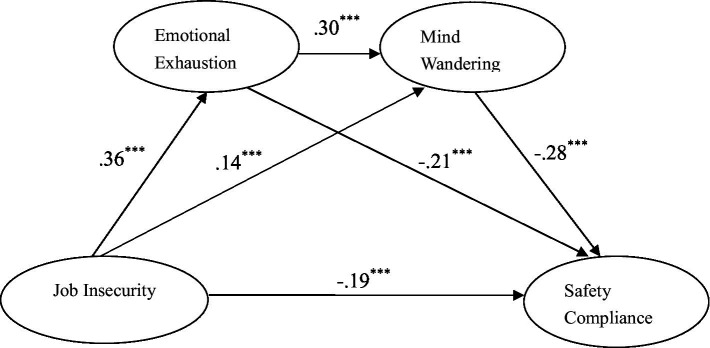
The chain mediating effect of miners’ emotional exhaustion and mind wandering (*N* = 447). ^***^p < 0.001.

Structural equation model analysis was used to test the multiple mediating effects of emotional exhaustion and mind wandering on job insecurity and safety compliance. First, Amos software was used to construct and analyzed the hypothesis model for the study. It is worth noting that, to test the model, we also constructed two competing models. The first competing model removed the path from emotional exhaustion to safety compliance, meaning that emotional exhaustion does not mediate the relationship between job insecurity and safety compliance but does mediate the relationship between job insecurity and mind wandering. The second competing model removed the path from job insecurity to mind wandering, meaning that mind wandering does not mediate the relationship between job insecurity and safety compliance but does mediate the relationship between emotional exhaustion and safety compliance. The two competing models had one less path than the study hypothesis model and thus were nested within the latter, which is convenient for performing structural equation model analysis for data fitting and comparison. The study hypothesis model fit the research data best (χ^2^/*df* = 4.85, CFI = 0.93, NNFI = 0.92, RMSEA = 0.054, SRMR = 0.051). Specifically, in Figure 2, job insecurity (*β* = −0.19, *p* < 0.001), emotional exhaustion (*β* = −0.21, *p* <. 001), and mind wandering (*β* = −0.28, *p* <. 001) all had negative effects on safety compliance; job insecurity had a positive effect on both emotional exhaustion (*β* = 0.36, *p* < 0.001) and mind wandering (*β* = 0.14, *p* < 0.001); and emotional exhaustion had a positive effect on mind wandering (*β* = 0.30, *p* < 0.001). Therefore, both emotional exhaustion and mental wandering play partial mediating roles in the effect of job insecurity on safety compliance; accordingly, H1, H2, H3, and H4 were all supported.

Second, given that the percentile bootstrap method of deviation correction can effectively reduce type II errors (Mackinnon et al., 2004), this study examined indirect effects by estimating the bootstrap 95% confidence interval. A total of 5,000 bootstrap samples were randomly selected. If the confidence interval does not contain 0, this indicates that the mediation effect is significant. The results are shown in Table 2. From Table 2, we can see that the total indirect effect value of emotional exhaustion and mind wandering on the effect of job insecurity on safety compliance among coal miners was −0.144 (accounting for 43.11% of the total effect), and the bootstrap 95% confidence interval was [−0.175, −0.117], i.e., did not contain 0. This means that emotional exhaustion and mind wandering have significant chain mediating effects.

**Table 2:** Bootstrap indirect effect test(*N* = 447).

Effect type	Effect value	Bootstrap SE	Bootstrap 95% CI	Relative mediating effect
Total indirect effect	−0.144	0.02	[−0.175, −0.117]	43.11%
Indirect effect 1	−0.075	0.01	[−0.096, −0.058]	22.45%
Indirect effect 2	−0.039	0.02	[−0.046, −0.031]	11.68%
Indirect effect 3	−0.030	0.02	[−0.037, −0.025]	8.98%

Indirect Effect 1 path = Job Insecurity → Emotional Exhaustion → Safety Compliance; Indirect Effect 2 path = Job Insecurity → Mind Wandering → Safety Compliance; Indirect Effect 3 path = Job Insecurity → Emotional Exhaustion → Mind Wandering → Safety Compliance.

## Discussion

### Effect of job insecurity on safety compliance among miners

In the context of the policy of de-overcapacity in the Chinese coal industry, through empirical investigation, this study examined the relationship between job insecurity, emotional exhaustion, mind wandering, and safety compliance among coal miners from three coal mines in Pingdingshan. Supporting previous work (Masia and Pienaar, 2010), the results showed that job insecurity had a negative effect on miners’ safety compliance, that is, the fear of unemployment can negatively affect miners’ safety compliance. This finding matches Maslow’s theory of hierarchy of needs (Maslow, 1943), indicating that having a secure job is a basic need of coal miners that can not be ignored. If this need is not secured, job insecurity will set in, which can seriously affect achievement motivation and work behavior among miners. At the same time, according to the social exchange theory (Emerson, 1987), when job security is lost, miners also lose their sense of trust in and belonging to the coal mine organization. Poor safety performance is the result of employees’ spontaneous behavior, in accordance with the principle of social exchange equity. The concept of job insecurity is seldom used in the study of coal mine safety management. This study not only expands the research field of job insecurity but also provides empirical evidence that can be used to promote good safety management practices in China.

### Mediating effect of emotional exhaustion

This study revealed the relationship between job insecurity and safety compliance among coal miners and demonstrated that job insecurity not only directly predicted miners’ safety compliance but also indirectly predicted miners’ safety compliance through emotional exhaustion. This indicates that job insecurity does not lead miners to concentrate on improving their performance and avoid unemployment but rather makes them suffer emotional exhaustion so that they thus become unwilling or insufficiently committed to following safety rules. The exploration of emotional exhaustion as the internal mechanism between job insecurity and safety compliance is the outstanding theoretical contribution of this study, which will enrich the empirical literature about social exchange theory and resource conservation theory. Miners’ concerns about job continuity and stability partially result from the overwhelming context of economic development and the national policy of de-overcapacity. In addition, it may partly reflect miners’ long dependence on coal for their livelihoods, organizations’ human resources management mechanism, which means miners have no right to choose their posts independently, and the reality that the miners’ voices are not taken seriously due to their marginalized status within the organization.

### Mediating effect of mind wandering

This study also showed that, as with emotional exhaustion, mind wandering also partially mediated the effect of job insecurity on safety compliance among coal miners. Specifically, job insecurity not only directly and negatively affected safety compliance but also indirectly and negatively affected safety compliance through mind wandering. Mind wandering is the uncoupling of the conscious state of attention. It is a common psychological phenomenon that all individuals experience repeatedly, every day (Barron et al., 2011). However, for coal miners who are engaged in high-intensity and high-risk work, mind wandering during work tasks represents a major potential safety hazard. Mind wandering is affected by many factors, such as physiology, the nature of the task, environment, and pressure (Li et al., 2018). Studies have shown that work stress significantly affects the state of consciousness among coal miners, especially the allocation of attention resources, thus increasing the incidence of mind wandering. This can reduce the ability to recognize safety signals, leading to operational errors, and finally causing unsafe behaviors (Li et al., 2018). For most coal miners, the perceived threat to their jobs is undoubtedly a huge psychological strain. If the threat is not rapidly removed, it can create a constant stressor that can cause serious psychological damage. According to the resource control theory, the amount of attentional resources available to an individual is fixed and limited. Executive control is a mechanism to prevent task-irrelevant thoughts from consuming the attentional resources required by the main task (Thomson et al., 2015). Job insecurity, as a type of continuous psychological stressor, constantly consumes the limited psychological resources of coal miners, making their executive control ineffective and forcing the allocation of attention resources. This can lead to mind wandering, whereby a miner may not to be effectively recognize and receive work safety information, resulting in poor safety compliance and increased operational errors.

### Chain mediating effects of emotional exhaustion and mind wandering

Emotions can have a significant impact on cognitive processes, such as perception, attention, memory, and reasoning (Tyng et al., 2017). Attention, a psychological state accompanied by various cognitive activities, can be easily affected by emotions, thus affecting an individual’s task performance (Murphy et al., 2012). This notion is supported by the results of the present study. We identified a strong link between emotional exhaustion and mind wandering, where both of them constitute an intermediate link in the influence path of Job Insecurity → Emotional Exhaustion → Mind Wandering → Safety Compliance; that is, emotional exhaustion and mind wandering had a chain mediating effect in the process of coal miners’ job insecurity, which affected their safety compliance. These results indicate that while emotional exhaustion and mind wandering played an independent mediating role in the relationship between job insecurity and safety compliance among coal miners, emotional exhaustion also indirectly affected safety compliance among coal miners by influencing mind wandering. This study of the chain-mediating effect of emotional exhaustion and mind wandering is a synthesis of resource conservation theory and resource control theory, which connected emotional exhaustion and mind wandering with job insecurity and revealed the influencing mechanism of coal miners’ safety behavior more comprehensively and deeply. Emotional exhaustion can make coal miners physically and mentally exhausted, so that they lack sufficient psychological resources to carry out adequate and appropriate cognitive processing of heavy and complex downhole tasks. In addition, it is difficult to steadily focus their attention on their current task, directly resulting in mind wandering. According to the decoupling hypothesis, frequent mind wandering severely depletes the resources responsible for executive control (Smallwood and Schooler, 2006), thus affecting employees’ compliance with safety rules. Therefore, emotional exhaustion can indirectly affect safety compliance of coal miners through mind wandering.

In conclusion, this study confirms that job insecurity indirectly affects coal miners’ safety compliance through chain mediating effects of emotional exhaustion and mind wandering. The job insecurity of coal miners is rooted in objective national strategic policies and is also influenced by their own subjective factors. This typical workplace stressor can have a range of serious psychological and behavioral effects on coal miners. According to the self-control model, the stress of executive self-control in response to job insecurity drains miners’ psychological resources, and long-time resource depletion can lead to emotional exhaustion (Wheeler et al., 2013; Whitman et al., 2014), which will be harmful to the self-control with respect to safety behavior (Schmeichel and Baumeister, 2004), and it can easily lead to a low level of safety compliance. Meanwhile, according to resource conservation theory, coal miners will follow the principle of conservation priority to prevent further loss of resources. Job insecurity is also associated with a variety of negative emotions, which can further lead to the internal consumption of limited psychological resources and easily result in emotional exhaustion, which makes miners unable to concentrate on processing the current target stimulus when working underground and ultimately seriously reducing their safety compliance.

### Practical implications

According to our finding that the job insecurity of coal miners directly negatively affects their safety compliance, coal production management departments and coal mine managers must pay close attention to coal miners’ mental state and take effective measures to reduce their insecurity. In view of the realistic social background that the coal mine closure wave caused by the de-overcapacity policy of coal production cannot be changed, it is necessary to provide psychological counseling and spiritual support for coal miners. Relevant departments and managers should try to stabilize coal miners’ mood, alleviate their anxiety and fear of unemployment, go deep into coal miners’ lives, care about their sufferings, listen to their hearts, understand their concerns, address their problems and relieve their life pressure and mental stress.

This study further found that job insecurity of coal miners can indirectly affect their safety compliance through the chain mediating effect of emotional exhaustion and mind wandering. Therefore, in strengthening coal mine safety management, the safety attention level of coal miners can be improved by reducing their emotional exhaustion. For example, the mental state of coal miners should be measured and screened regularly. For miners with high levels of emotional exhaustion, it is possible to reduce their emotional exhaustion and increase their safety attention by patiently educating and genuinely caring for them through their team leader or colleagues. The human resources management department of a coal mine can also arrange professional psychological counselors to provide emotional group counseling for miners to enhance their emotional regulation ability, reduce emotional exhaustion, and improve safety attention.

### Research limitations and future prospects

There are some shortcomings and limitations to this study, although it also provides a new breakthrough for future research. First, this study only included coal miners from the city of Pingdingshan, China, and the sample area is relatively localized, which weakens the scope of wider applicability of the research conclusions. Follow-up studies with a larger sample size would be useful and, if possible, a larger national survey could be conducted to determine the causal link between job insecurity and safety compliance among coal miners. Second, the data in this study came from miners’ self-reporting. Although some measures have been taken to avoid common method bias, its influence on the research conclusions cannot be completely excluded. Follow-up studies could take a flexible approach to the collection of safety behavior data, through a combination of leaders’ evaluations and miners’ self-evaluations to further avoid the influence of common method bias. Third, only a few variables were explored in this study, and the theoretical model constructed was also relatively simple. Subsequent research could further enrich this model by including miners’ psychological capital, emotional intelligence, social support, organizational justice, work engagement, job burnout, work–family conflict (WFC), etc., to more comprehensively explore the mechanisms influencing coal miners’ safety performance. For example, regarding the role of organizational justice, Wang et al. (2015) have found that organizational justice plays a moderating role in the relationship between job insecurity and job performance. Similarly, De Angelis et al. (2021) have provided support to the role of organizational justice in decreasing the association between job insecurity and job performance among employees of an Italian multiservice social cooperative. In addition, it is necessary to mention that, as participation occurred in a work context, it would be possible that participants were less frank than they would have been in another context. After all, miners are a vulnerable group. Being cautious of jeopardizing their jobs by openly reporting their work experiences, participants may have, deliberately or unwittingly, provided socially desirable responses. To some extent, this will affect the accuracy of the research results.

### Conclusion

Using Maslow’s theory of a hierarchy of needs, the strength model of self-control, and the conservation of resources theory, we provided a theoretical framework and empirical support for a link between job insecurity and safety compliance among coal miners. The results showed that job insecurity not only directly but also indirectly harms coal miners’ safety compliance *via* emotional exhaustion and mind wandering. Specifically, emotional exhaustion and mind wandering played a chain mediating effect in the relationship between job insecurity and miners’ safety compliance. This study helps to advance our understanding of the internal mechanism of coal miners’ job insecurity and how this affects individual safety performance. It also provides empirical evidence for managers to effectively intervene in coal miners’ safety performance.

## Data availability statement

The raw data supporting the conclusions of this article will be made available by the authors, without undue reservation.

## Author contributions

The author confirms being the sole contributor of this work and has approved it for publication.

## Conflict of interest

The author declares that the research was conducted in the absence of any commercial or financial relationships that could be construed as a potential conflict of interest.

## Publisher’s note

All claims expressed in this article are solely those of the authors and do not necessarily represent those of their affiliated organizations, or those of the publisher, the editors and the reviewers. Any product that may be evaluated in this article, or claim that may be made by its manufacturer, is not guaranteed or endorsed by the publisher.
